# Typology of Substance Use Disorder Based on Temperament Dimensions, Addiction Severity, and Negative Emotions 

**Published:** 2018-07

**Authors:** Mohammad Bagher Saberi Zafarghandi, Hamid Khanipour, Seyed Mojtaba Ahmadi

**Affiliations:** 1 Department of Addiction, School of Behavioral Sciences and Mental Health (Tehran Institute of Psychiatry), Iran University of Medicine Sciences, Tehran, Iran.; 2 Institute of Educational, Psychological and Social Research, Psychological Studies Group, Kharazmi University, Tehran, Iran.; 3 Department of Clinical Psychology, School of Medicine, Student Research Committee, Shahid Beheshti University of Medicine Sciences, Tehran, Iran.

**Keywords:** *Addiction*, *Negative Emotion*, *Substance Use Disorder*, *Temperament*, *Typology*

## Abstract

**Objective:** Individuals with substance use disorder are not homogeneous as we might regard. Thus, this study was conducted to present a novel classification of substance use disorder based on temperament, addiction severity, and negative emotions.

**Method** **:** In this correlation study, Temperament and Character Inventory, Addiction Severity Index, Aggression Subscale of MMPI-2, Depression, Anxiety, Stress Scale, Emotional Schema Questionnaire, and Psychosocial Checklist were used for data collection.

**Results: **The cluster analysis of 324 individuals with substance use disorder explored 4 subtypes. Subgroups were named based on the main features as emotionally distressed, constitutional, nonconformist, and impulsive. Significant differences were found among groups in emotional schemas, history of mental disorder in the family, rate of relapse, and history of imprisonment.

**Conclusion: **It seemed that temperament dimensions, addiction severity, and negative emotional states were valid components in classifying individuals with substance use disorder.

There have been many variations exist in the nature, but our mind prefers to simplify these natural variations to homogenous categories. We always categorize substance use disorders based on observable indicators, including the type of substances that have been abused (1). This type of classification is very useful for formal diagnosis, but it is not sufficient to depict the whole picture of problems that a patient with substance use disorder experience. Another major problem is that there are many differences between substance users who are addicted to the same kind of substance (2). Changing the systems of classification of mental disorders from categorical to dimensional have been one of the reasonable responses to these kinds of problems.

In line with the idea of heterogeneity among substance and alcohol users, some efforts have been made to identify subgroups of individuals with substance use disorder by statistical methods, such as cluster analysis and factor analysis (3-13). For example, it has been shown that 2 types of clusters exist in alcohol abusers, which are called type A (late onset, low severity) and type B (early onset, high severity) that differ based on onset of alcohol abuse and comorbidity of emotional problems and history of delinquency and antisocial behaviors (2, 3, 10).

One of the advantages that have been demonstrated for these types of studies is that we can properly match patients with appropriate pharmacological or psychological interventions (3-5).

The main question that shapes the classification of substance use disorders is as follows:

Which risk factors should be selected for each classification? 

The main question that shapes the classification of substance use disorders is as follows:

Which risk factors should be selected for each classification?

Reviewing studies that used cluster analysis in substance use disorders revealed that temperament, addiction severity, and positive and negative emotions were the highly repetitive factors that have been used in classification studies (3, 6, 7, 8, and 13). Temperament was conceptualized as a biological basis of personality that expresses its effects through different behavioral responses to stimuli, such as danger or rewards cues (14). In psychobiological theory of temperament and character, temperament dimensions have been considered as novelty seeking, harm avoidance, and reward dependence. Novelty seeking was assumed as a brain behavioral system that predispose people to be more impulsive toward new stimuli and less tolerant against frustrations, and it was associated with substance abuse (14). Harm avoidance is also one of the temperament dimensions that have a role in negative feelings and avoidance behaviors and involves several psychological mechanisms, including worry, shyness, fatigability, and fear of uncertainty (14). Reward dependence is another temperament dimension. Individuals who obtain a high score in reward dependence are eager to obtain social and physical rewards, are more gregarious, and are also vulnerable toward some psychopathologies, including pathological gambling, alcoholism, and substance use disorders (14). 

 Addiction severity or the severity of problems related to substance use disorder have been regarded as a main factor for classifying different types of substance use disorders up to now (8, 10). Addiction severity was measured by some indices including problems in domains of family, job, physical health, mental health, and drugs. Substance abusers can be classified into 2 types: one with high and the other with less severity. Also, negative emotions were robust differential factors in classifying substance abusers (8, 10, and 11). Furthermore, negative emotional states, such as depression, anxiety, and aggression, significantly distinguished different types of substance use disorders (2, 3, 6, 7, 10, and 11). Many substance abusers may show comorbid antisocial behaviors, and some typology studies have found different subtypes among substance abusers with offending behaviors based on personality traits and addiction severity (9, 10). Although these types of typology studies have been conducted more on individuals with alcohol use disorder, less classification studies have been conducted on other substance use disorders, and the existing typologies are only based on the severity of addiction and temperament dimension. Furthermore, less is known about the role of other contributing factors such as negative emotional states as a clustering factor. The aim of this study was to separate individuals with substance use disorders into subgroups based on temperament dimensions, addiction severity, and negative emotions. Classifying a heterogeneous group of individuals into smaller categories has the benefit of identifying groups of people who may have different courses of symptoms and different treatment responses. The first aim of this study was to investigate the classifications of individuals with substance use disorder based on temperament components, addiction severity, and negative emotions, including depression, anxiety, stress, and aggression. In addition, the second aim of this study was to compare identified groups in emotional schemas and some psychosocial indices, including history of substance abuse in the family, relapse rate, history of childhood abuse, imprisonment, and history of mental disorder in the family.

## Materials and Methods

To explore subgroups in individuals with substance use disorder, we adopted a correlational method for data collection. In this study, Temperament and Character Inventory (15), Depression, Anxiety and Stress Scale (16), Aggression Subscale of Minnesota Multiphasic Personality Inventory-2 (17), Emotional Schema Questionnaire (18), and Addiction Severity Index were used (19).

Participants were 324 individuals with substance use disorder who received medical and psychological services in substance use treatment centers in Tehran. Participants were outpatients from methadone maintenance therapy (MMT) clinics and those who were under therapeutic interventions in a residential program, which was held in South Tehran Health Center. Convenient sampling method was used to select the participants. Inclusion criteria were as follow: (a) diagnosis of substance use disorder; (b) age 18 to 60 years; (c) at least having two-year history of substance use disorder; and (d) having been enrolled in a drug treatment program. Exclusion criterion was a diagnosis of psychotic disorder or any type of cognitive disorders. Informed consent was gained prior to the study from all of the participants.

*Measures*

1. Temperament and Character Inventory: It is a well-known questionnaire for assessing personality traits. It consists of 240 questions that assess 3 dimensions of temperament and 4 dimensions of character (15). In this study, we used 3 dimensions of temperament (i.e., novelty seeking, harm avoidance, and reward dependence). Factor structure of this inventory have been confirmed in an Iranian sample (20). Also, results of assessing the internal consistency of these dimensions in a group of individuals with psychiatric disorder showed that Cronbach’s coefficient for novelty seeking, harm avoidance, and reward dependence was 0.86, 0.75, and 0.67, respectively (20).
2. Depression, Anxiety and Stress Scale: It has 21 statements that screen the severity of emotional distress, including depression, anxiety, and stress (16). The questions of this scale are rated on a 4- point Likert scale. Factor structure of this scale in a sample of Iranian undergraduate students confirmed the existence of 3 factor. Cronbach’s alpha for this scale for depression, anxiety, and stress was 0.87, 0.82, 0.81, respectively (21).
3. Aggression Subscale of MMPI-2: This subscale of Minnesota Multiphasic Personality Inventory (MMPI-2) consists of 22 statements that are rated on yes or no format (17). It has been validated on different groups of individuals, including those with substance use disorders. This inventory has been used in different studies to distinguish the subtypes of substance abuse (8).
4. Emotional Schemas Questionnaire: In this study, some subscales of this questionnaire (18), including rumination, non-acceptance of feeling, blaming others, expressing emotions, worry, and rational thinking, were used. The questionnaire assesses how people deal with their different feelings. Every statement is scored in a Likert type scale from 1 (very untrue of me) to 7 (very true of me). The reliability and construct validity of this questioner have been confirmed in a sample of Iranian undergraduate students (22). Cronbach’s alpha for the subscales were reported to be 0.66 to 0.88 (22).
5. Addiction Severity: This is a screening tool implemented as a semi-structured interview (19). Questions were asked from participants. Then, they were calculated, and a severity index was delineated for each aspect of problems. Some indices of addiction severity index consisted of problems in such domains as family problem, work-related problems, mental health problems, and medical problems were administered in this study. These indices completely corresponded to the inclusion criteria and were used for diagnosis of substance use disorder according to DSMV. Psychometric studies revealed that internal consistency (Cronbach’s alpha) for almost all the subscales of this instrument was greater than. 70, and its concurrent validity with other indices of addiction severity was moderate in a homeless group (23). This instrument has been applied for Iranian methamphetamine users, and it showed a sensitive index that could reveal the possibility of change through psychotherapy (24).
6. Psychosocial Screening Checklist: This checklist includes some questions about history of substance use disorder, mental disorder in family members, and past records of imprisonment, suicide attempt, and childhood abuse, and the rate of relapse. These questions were rated in a yes/no format, were designed to compare groups, and were extracted after cluster analysis. Another goal of using these questions was to examine the validity of extracted groups.

Hierarchical cluster analysis with complete linkage was used as a method to derive subgroups of individuals with substance use disorder. It is a statistical method that sorts cases in homogenous groups. Hierarchal cluster analysis was based on calculating the distance between samples and explore groups in several steps by delineating dissimilarity between groups (25). The method of complete linkage was the most popular means for cluster analysis, which explored groups based on the maximum of the pair of dissimilarities in each case and yielded cluster with enough number of individuals (25). Also, we used analysis of variance (ANOVA) and chi-square to compare the derived subgroups.

## Results

From 324 individuals, 314 were male and 10 female. In total, the age distribution of participants was as follows: ¬20 (5%) in the age group of 18 to 25 years, 84 (21%) in 25 to 35, 124 (32%) in 35 to 45, and 96 (24.5%) in the age group of 45 to 60 years. From the participants who responded to the item about academic status, 156 had secondary school degree, 94 had a high school diploma, 29 associate degree, and 3 had postgraduate degree. 

The most common drug abused as a first drug was opium (n = 88, 22.4%). Frequency of other drugs, including heroin, Iranian crack, cocaine, methylphenidates, hallucinogens, and alcohols, was 14.3% (n = 56), 3.3% (n = 13), 4.6% (n = 18), 14.3% (n = 56), 6.6% (n = 26), and 10.2% (n = 40) respectively.

 To extract subgroups, such variables as temperament dimensions, addiction severity indices, negative emotions, and aggression were used. Running cluster analysis on data revealed 4 subgroups. Distance table and dendrogram were used to identify these subgroups from cases. The final cluster centers for clustering variables are demonstrated in Table1.

The frequency of groups from 1 to 4 was 82 (25%) for group 1, 69(21%) for group 2, 134 (38%) for group 3, and 39 (16%) for group 4, respectively. Characteristics of groups in clustering variables are presented in Table 2. 

The mean and standard deviation scores of 4 subgroups in temperament dimensions, negative emotional states, aggression, and indices of addiction severity are demonstrated in table 2.

Finding of analysis of variance revealed that the differences in all the comparing variables were significant. Post hoc analysis by Tukey test showed that novelty seeking of group 2 was significantly higher than groups 1 (mean differences = 1.77, p<0.001) and 3 (mean differences = 1.33, p<0.001). Harm avoidance in group 2 was higher than group 1 (mean differences = 2.26, p<0.001), groups 3 (mean differences = 0.706, p<0.026) and 4 (mean differences = 1.85, p<0.001). Reward dependence of group 3 was significantly lower than groups 1 (mean differences = -0.78, p = 0.001) and 4 (mean differences = -0.908, p<0.001). Also, the mean score of group 1 in depression, anxiety, and stress was significantly higher than groups 2 (p<0.001) and 3 (p<0.001), but there was not a significant difference between groups 1 and 4 in these 3 negative emotional states (p>0.5). Although the mean score of group 4 in aggression was higher than other groups, only the difference between group 3 and 4 was significant (p = 0.034). Severity of family problems, job problems, and physical health in group 4 was higher than other groups. However, in problems related to mental health, the differences were not significant between group 1 and group 4 (mean difference = -0.77, p = 0.153). 

A significant difference was found between groups in some of the emotional schemas, including rumination (F = 5.013, p = 0.002), worry (F = 8.69, p = 0.001), and blaming others (F = 4.75, p = 0.003). Post hoc analysis revealed that members of group 1 had higher mean score in rumination than group 2 (mean difference = 2.39, p = 0.004). Also, post hoc analysis showed a significant difference between group 1 and 2 in worry (mean difference = 2.43, p<0.001) and blaming others (differences of means = 1.86, p = 0.002). In sum, the mean score of group 1 and 4 in negative emotional schemas was very close to one another, and there was no significant difference between the 2 groups. 

The status of groups in some aspects of psychosocial history is presented in Table 3. Comparisons of groups in some aspects of psychosocial history revealed that the history of childhood abuse, history of suicide attempt, history of imprisonment, and relapse rate were significantly higher in group 3 than other groups (p<0.001). However, there were no significant differences between groups in history of substance abuse in family members. Finally, comparison between groups showed that the frequency of mental disorder in family members of group1 was significantly higher than other groups (n = 34, p<0.01).

**Table1:** Final Cluster Solution Centers Based on Temperament, Addiction Severity and Aggression

**Clusters**	**1**	**2**	**3**	**4**
Family problem	7.14	9.78	10	8.67
Job problem	8.41	11.23	10.45	8.79
Mental health	3.16	10.66	11.14	6.32
Physical health	1.31	5.62	5.60	2.85
Novelty seeking	4.26	4.93	2.79	5.08
Harm avoidance	4.59	4.04	3.66	5.63
Reward dependency	3.76	4.64	4.14	3.80
Aggression	10.86	8.82	14.71	3.63

**Table 2:** Summary of Statistics for Comparing Subgroups in Temperament, Addiction Severity and Negative Emotions

	**Group1** **M(SD)**	**Group2** **M(SD)**	**Group3** **M(SD)**	**Group4** **M(SD)**	**F**	**sig**
Novelty seeking	3.84(2)	5.6(1.97)	4.30(2.06)	4.26(2.01)	42.97	0.001
Harm avoidance	3.31(1.33)	5.58(1.96)	4.87(1.71)	3.72(1.77)	79.58	0.001
Reward dependency	4.51(1.64)	4.20(1.58)	3.72(1.35)	4.6(1.56)	6.45	0.001
Depression	9.1(4.21)	4.46(3.31)	6. 09(4.1)	8.70(4.34)	18.02	0.001
Anxiety	7.71(3.34)	4.17(3.61)	5.74(4.2)	7.45(3.50)	11.08	0.001
Stress	11.11(4.69)	5.15(3.89)	7.47(4.84)	10.10(4.58)	19.38	0.001
Aggression	10(4.20)	8.46(3.7)	8.47(4.42)	10.55(3.54)	4. 41	0.005
Family problems	9.25(1.67)	9.76(1.47)	7.56(2.14)	10.96(1.64)	45.90	0.001
Job problems	10.91(1.59)	9.64(1.86)	8.40(0.75)	12.75(2.05)	111.19	0.001
Physical health	5.71(1.58)	4.62(1.15)	1.55(1.44)	6.43(1.53)	209.10	0.001
Mental health	11.05(1.70)	9.09(1.37)	3.83(2.17)	11.82(1.92)	349.72	0.001

**Table 3:** Summary of Statistics for Comparing Subgroups Based on Psychosocial History

**Groups**		HMDF*						HCA*						HSA*						HSUF*							HI*						RR*							
	**Yes**			**no**		**X** ^2^		**yes**		**no**		**X** ^2^		**yes**		**no**		**X** ^2^		**yes**		**no**		**X** ^2^		**yes**			**no**		**X** ^2^			**1-2**		**3-4**		**5**		**X** ^2^
1	39		41				71		11				22		60				43		36				21			60				17			25		38			
2	15		49		20.3		59		8		13.7		7		60		40.7		33		34		4.5		13			54		8.2		25			15		23		13.1	
3	34		74				90		38				37		95				54		76				84			48				25			23		65			
4	19		19				33		5				17		21				14		23				15			23				7			8		21			

*History of Mental Disorder in Family, *History of childhood abuse, *History of suicide attempt, *History of substance use in family members, * History of Imprisonment, * Relapse rate

## Discussion

The aim of this study was to assess and classify individuals with substance use disorder based on some fundamental factors that have been hypothesized to be valid indictors for classification of substance abusers beyond known factors, such as type of drug. Findings of cluster analysis showed 4 different subgroups that had different mental and behavioral profiles. Also, there were major differences between these groups in domains related to addiction severity. Addiction severity in groups 1 and 2 were less than groups 3 and 4. The typology that was derived in this study is consistent with some other typologies in alcohol and substance use disorder (8, 10). In all the cluster analyses, it seemed that severity of problems in such domains as family life, job, physical, and mental health was a robust factor that could be used in separating individuals with substance use disorders. Thus, in the treatment of substance use disorders, attending to severity of problems need to be considered and type of treatment should be tailored for each group based on domains related to addiction severity. 

Group1 was characterized by the highest level of emotional problems, including depression and anxiety, and they had the worst score in mental health domain of addiction severity. Also, the frequency of mental disorders in family members of individuals who belonged to this group was outstandingly higher than other groups. It seems that the reason they abused substance was not seeking pleasure because they had the lowest score in novelty seeking among temperament dimensions, which was related to reinforcement and pleasure. According to their pattern of emotional problems, severity of substance dependence problems, and temperament, it could be inferred that this group was emotionally distressed. It seemed that they used substance primarily for self-medication, i.e., alleviating their negative emotional experiences. Comparison of groups revealed that negative emotional schemas, specially worry and rumination, was used more by members of group 1. This finding corroborates this idea that members of this group were emotionally distressed people who used drugs primarily to overcome depression, anxiety, or other types of emotional problems. The relationship between emotional disorders, 

such as major depressive disorder and general anxiety disorder, and substance abuse or dependence has been demonstrated in some studies (26, 27). So, untreated emotional disorders may lead to a full-blown substance use disorder. 

Group 2 showed a typical profile of individuals with substance use disorder who had personality vulnerability for such types of problems. They had the lowest score in indices related to addiction severity, but their scores in temperament dimensions, which were related to addiction, including novelty seeking and harm avoidance, were the highest among other groups. They were prone to substance abuse because of internal predisposition, so this group could be called as constitutional substance users group. Also, in other cluster analysis studies in substance use disorders, some subgroups that had the same clinical profile, had been identified, including sensation seeker group or genetically vulnerable subgroup (8, 10, and 13).

The main features of the members of group 3 were a pattern of the lowest score in reward dependence, partially higher score in novelty seeking, and less severity of problems related to substance dependence. The number of individuals in this group was more than other groups. In addition, the frequency of suicide attempt, history of imprisonment, and abuse (physical, sexual or emotional) in this group were higher than other groups. Profile of people with low score in reward dependence showed that they tend to be nonconformists and introverts, with low interest in common social values (14). The characteristics of this group was very similar to alcoholic and introverted/hopeless types of substance abusers (2, 3, 7, and 10). According to this type of temperament pattern and severity of problems related to substance dependence and psychosocial indices, this group could be called as a non-socialized or nonconformist group. This type of substance users is very similar to anomic type of social pathology that some sociologists have mentioned (28). According to our results, it could be suggested that a mixture of factors, including internal insensitivity toward common social values and childhood maltreatment, may have a role in most substance use disorders. It could be said that the non-socialized group have some problems in socialization process that leads to violating accepted social rules and they show deviant behaviors such as addiction. 

The main features of individual members of group 4 were obtaining highest scores in all the domains related to addiction severity, the highest score in reward dependence, the highest score in aggression, and the lowest score in harm avoidance. These patterns of features are much closer to impulsive or sociopathic subtypes of substance abuse that were identified in previous typology study (9, 10, and 12). 

 According to the findings of this study, individuals with substance use disorder had a tendency to drugs for different reasons. In group1, the main reason was comorbid mental disorder and they probably used illicit substance to alleviate depression or anxiety. The main factor in drug use in group 2 was seeking more pleasure and excitement. The main reason for drug use in group 3 was the less ability to incorporate social values. Finally, it could be suggested that the main reasons of group 4 were impulsivity and deficiency in inhibiting or blocking clues associated with rewards. 

Also, comparison of groups in psychosocial variables, including history of suicide attempt, rate of relapse, history of imprisonment, frequency of mental disorder, and substance use disorder in family members, and history of childhood abuse confirmed the validity of clusters. Rates of relapse was different among the 4 groups and had the highest degree in group 3. The higher rate of relapse in group 3 may be explained by their score in temperament dimensions or their past history of imprisonment or childhood abuse. Also, there were significant differences between the 4 groups in emotional schemas. Our findings revealed that individual members of groups who had severe problems in substance dependence indices used more negative and maladaptive emotional schemas, such as rumination, guilt, blaming others, and worrying. It could be concluded that negative and maladaptive emotional schemas may be a contributing factor in exacerbating addiction severity. This pattern of emotional schemas in the 4 subgroups showed that for an optimal intervention, we need to adapt therapeutic intervention for different groups. It could be suggested that people in group 1 need pharmacotherapy and psychological treatment, such as cognitive behavioral therapy to overcome comorbid negative emotional states. Priority of treatment for group 2 could focus on motivation- enhancing strategies that facilitate a cognitive dissonance situation, which may lead to change. Members of group 3 need more environmental enrichment and supportive strategies to encourage their social interests, and members of group 4 need such strategies as social problem solving and anger management to alleviate impulsivity.

## Limitation

This study had some limitations that should be considered in interpreting the findings. First, participants were assessed by self-report measures, which may not be good enough to obtain true data. Second, our participants used different types of drugs. Third, only 3% of the sample were female, so the results of this study could hardly be generalized to women with substance use disorder. Fourth, a sample of participants was selected from treatment centers, and this group might have been psychologically different from the group that had ever referred for treatment. Fifth, the participants’ diagnosis was not checked by an empirically based method. Further studies should cover this shortage by applying good psychometric measures. Sixth, this study was conducted in a large city, and this sample was not representative of the rural population. The typology for opioid abusers may be different from stimulant abusers. Thus, further research is needed to investigate whether typology derived in these studies was relevant for individuals with substance use disorders who solely used one type of drug or not. Also, further studies could examine which types of psychosocial intervention were more appropriate for subgroups, which was explored in this study. 

## Conclusion

The results of this study revealed that there are various forms of substance use disorders that could be separated based on temperament dimensions, addiction severity, and negative emotional states. We reached 4 classes of substance use disorder. Furthermore, they differed in emotional schemas and rates of relapse, which corroborated the identified typologies. Contribution of our study that could distinguish our findings from those of previous typologies studies are as follow: (1) in this typology study, we tested a new classification based on more psychological factors, including temperament and negative emotional states; (2) our study also revealed a 4-cluster typology, which had distinctive profiles in domains such as psychosocial history and emotional schemas; (3) and we reached 4 subtypes which were named based on the main features as emotionally distressed, constitutional, non-conformist, and impulsive. It seemed that people with substance use disorder were more from non-conformist group.
